# AGING IN SUB-SAHARAN AFRICA

**DOI:** 10.1093/geroni/igae098.1712

**Published:** 2024-12-31

**Authors:** Jennifer Stanley, JohnBosco Chukwuorji, Jennifer Stanley

**Affiliations:** Chair; Co-Chair; Discussant

## Abstract

Aging in sub-Saharan Africa is a relatively neglected area of research despite the increasing proportion of the population aging. This collection of papers highlights how the varied cultures in sub-Saharan Africa shape development in later adulthood and emphasizes the critical need for research on aging to consider the sociocultural context. First, Chukwuorji and colleagues focus on how health behaviors and self-esteem explain the association between social support and successful aging among Nigerian retirees. Second, Porter and colleagues present a collaboration between scientists and older adults in knowledge generation. They find that access to both social and physical environmental elements are necessary to promote physical activity in their communities. Third, Burns and colleagues explore how place and gender interact when examining disability prevalence among older adults in Ghana. Fourth, Silverstein and colleagues report that culture and religion play an important role in shaping how palliative care is perceived. For example, a cultural norm for medical secrecy and a hesitancy to talk about death present unique barriers for palliative care. Fifth, in a qualitative study examining how older chronically ill Nigerians cope with their diagnosis, Mahmoud and colleagues report that older Nigerians do not want individuals outside their close family circle to know that they are ill. Individuals conceal their illness because they fear discrimination and want to maintain normalcy when interacting with others. All the papers presented in this symposium highlight the importance of considering the sociocultural context to understand the aging experience.

